# Using Association Rules to Obtain Sets of Prevalent Symptoms throughout the COVID-19 Pandemic: An Analysis of Similarities between Cases of COVID-19 and Unspecified SARS in São Paulo-Brazil

**DOI:** 10.3390/ijerph21091164

**Published:** 2024-09-01

**Authors:** Julliana Gonçalves Marques, Bruno Motta de Carvalho, Luiz Affonso Guedes, Márjory Da Costa-Abreu

**Affiliations:** 1 Department of Informatics and Applied Mathematics, Federal University of Rio Grande do Norte, Natal 59078-970, Brazil; bruno@dimap.ufrn.br; 2 Department of Computer Engineering and Automation, Federal University of Rio Grande do Norte, Natal 59078-970, Brazil; affonso@dca.ufrn.br; 3 Department of Computing, Sheffield Hallam University, Sheffield S9 3TY, UK; md0948@exchange.shu.ac.uk

**Keywords:** COVID-19, apriori, symptoms, association rule mining, symptom patterns

## Abstract

The efficient recognition of symptoms in viral infections holds promise for swift and precise diagnosis, thus mitigating health implications and the potential recurrence of infections. COVID-19 presents unique challenges due to various factors influencing diagnosis, especially regarding disease symptoms that closely resemble those of other viral diseases, including other strains of SARS, thus impacting the identification of useful and meaningful symptom patterns as they emerge in infections. Therefore, this study proposes an association rule mining approach, utilising the Apriori algorithm to analyse the similarities between individuals with confirmed SARS-CoV-2 diagnosis and those with unspecified SARS diagnosis. The objective is to investigate, through symptom rules, the presence of COVID-19 patterns among individuals initially not diagnosed with the disease. Experiments were conducted using cases from Brazilian SARS datasets for São Paulo State. Initially, reporting percentage similarities of symptoms in both groups were analysed. Subsequently, the top ten rules from each group were compared. Finally, a search for the top five most frequently occurring positive rules among the unspecified ones, and vice versa, was conducted to identify identical rules, with a particular focus on the presence of positive rules among the rules of individuals initially diagnosed with unspecified SARS.

## 1. Introduction

The dynamics of the COVID-19 pandemic have set it apart from previous pandemics. After enduring multiple waves of infection over three years, the World Health Organization (WHO) officially declared the end of the state of a public health emergency on 5 May 2023 [1]. However, COVID-19 persists and continues to affect the global population. Various studies have enhanced understanding of the virus and its variants, shedding light on various factors influencing disease diagnosis, with symptoms being the most recognisable to the public.

Several different symptoms associated with COVID-19 have been documented [2,3,4,5]. Moreover, these symptoms can vary across different variants [6,7], implying that the symptom patterns used to identify one variant may not be applied to others. Considering that symptom dynamics can affect the diagnosis of COVID-19 differently, association rule mining can play a significant role in uncovering symptom patterns within disease data.

There are different ways to discover patterns in datasets in machine learning (ML). As an example, association rule mining (ARM) refers to rule-based machine learning methods that seek to recognise relationships between items, identify frequent patterns, and facilitate the exploration of associations and correlations among items in datasets [8,9,10]. Regarding ARM approaches, the Apriori algorithm is the most classical for mining frequent item sets along with FP-growth, and both are widely applied to large datasets.

The techniques have been reported in the literature to find symptoms patterns in COVID-19 data, as in the study proposed by [11] that aimed to discover symptom patterns and overall symptom rules by observing data in different scenarios. They used a dataset containing information on 1560 patients, generating 71 rules using the Apriori algorithm. The results showed that the most frequent symptoms were fever (67%), followed by cough (37%), malaise/body soreness (11%), pneumonia (11%), and sore throat (8%), whereas 1% of the population presented with severe symptoms, such as septic shock, respiratory distress syndrome, and respiratory failure.

Another analysis applied the Apriori algorithm to discover symptom patterns in COVID-19 patients [12]. They collected a dataset containing 2875 cases and 32 features from hospitals in Iran from March 2020 to March 2021. The experiments identified apnea as the most common symptom (72%), followed by cough (64%), fever (59%), weakness (18%), myalgia (14.5%), and sore throat (12%). Other symptoms were also reported in 1–10% of cases, such as nausea, diarrhea, and respiratory distress syndrome.

However, most works aimed to perform COVID-19 diagnosis using ARM algorithms, while others proposed exploring the relation of COVID-19 with mortality and chronic diseases, as in [13], which used the rule-based FP-growing algorithm to predict COVID-19. Their dataset consisted of symptoms and other factors from 5434 cases. The results showed that six factors appeared to be mainly responsible for COVID-19 infection: breathing problems, fever, dry cough, sore throat, and travel abroad.

The authors of [14] proposed using the Apriori algorithm as a novel approach to predict COVID-19 symptoms. Experiments with clinical data generated nine rules and pointed out that fever, cough, difficulty breathing, and sore throat were the most common outcomes of infected individuals’ rules. In addition to diagnosis, [15] proposed patient treatment using the Apriori algorithm. Experiments were conducted on 49 confirmed COVID-19 deaths diagnosed, and symptom and imaging features were collected. The most frequent symptoms were difficulty breathing, fatigue, cough, chills, gastrointestinal symptoms, muscle aches, and tachycardia. The association rule analysis also concluded that factors such as immunodeficiency, hypertension, and coronary heart disease associated with COVID-19 increased the chance of death.

Time-series modelling and association rule mining were applied in [16] with the aim of identifying meaningful prognostic factors and predicting the number of cases required for efficient COVID-19 crisis management. A dataset of 3685 individuals containing 35 features was used, and 595 rules were extracted. The results showed, among other conclusions, that worsening cases requiring referral to the hospital ward were associated with patients admitted with pregnancy, metabolic syndrome, and age greater than 65 years. Using the H-Apriori algorithm, ref. [17] developed a mortality prediction model for cardiovascular patients with COVID-19 patterns using association rule mining. The experiments used a dataset of 3971 patients, and 57 rules were generated. According to the results, a significant number of patients with fever, sore throat, and cough were associated with high cholesterol, diabetes, and obesity. In addition, chest discomfort or pain was more prevalent among stroke patients, and those with this symptom were more likely to have diabetes and hypertension.

As can be seen, most of the work compared different groups regarding the clinical presentation and consequences of COVID-19. However, only individuals who were positive for COVID-19 participated in the experiments, with none of them investigating, from positive disease patterns, the presence of COVID-19 in initially unspecified SARS individuals.

In this paper, the use of the association rule-based technique to analyse the set of prevalent symptom rules of positive and unspecified SARS patients to discover similarities between the symptom rules is proposed. Using the Brazilian Severe Acute Respiratory Syndrome (SARS) database, data from the first two years of the pandemic of COVID-19, specifically cases from July 2020 and March 2021 in São Paulo State, Brazil, periods with the highest number of infection cases in the state, will be analysed.

The remainder of this paper is organised as follows: Section 2 presents the dataset used, offering a succinct explanation of the COVID-19 lineages in São Paulo, along with the conceptualisation of the technique applied in the experiments. In Section 3, the results are presented, and Section 5 discusses the findings and proposes future directions.

## 2. Materials and Methods

### 2.1. Data Acquisition

For the experiments, COVID-19 and unspecified SARS cases from 2020 (1,007,052 individuals) and 2021 (976,633 individuals) were obtained from the SARS database available at [18], a publicly accessible database related to the Brazilian health situation. These periods were chosen because the first two years of the pandemic registered the highest number of infections and deaths worldwide, especially in Brazil. In addition to symptom variables, the datasets contained social demographics, risk factors, comorbidity, and laboratory findings for all states, totalling 80 variables. A description of all database variables can be found in [19].

### 2.2. Feature Selection

To perform the experiments, COVID-19 and unspecified SARS cases in the state of São Paulo were selected. Due to the large number of inhabitants and tourists, it is believed that the state represents the most dynamic COVID-19 scenario in the country, making the data a substantial source of information for the experiments. Thus, the peak periods for the number of infection cases in both years were used. In 2020, the period with the most cases registered was July, and in 2021, it was March [20]. Next, the 12 symptoms used for the experiments are described.

1. ABDOMINAL PAIN (ABDPAIN): whether the patient presented with abdominal pain.
2. COUGH: whether the patient presented with a cough.
3. DIARRHOEA: whether the patient presented with diarrhoea.
4. DYSPNOEA: whether the patient presented with dyspnoea.
5. FATIGUE: whether the patient presented with fatigue.
6. FEVER: indicates whether the patient presented with fever.
7. LOSS OF SMELL (LOSSM): whether the patient presented with a loss of smell.
8. LOSS OF TASTE (LOSST): whether the patient presented with a loss of taste.
9. RESPIRATORY DISTRESS (RESPDESC): whether the patient presented with respiratory distress.
10. SATURATION: whether the patient presented with low oxygen saturation ($O_{2}<$ 95%).
11. THROAT: Indicates whether the patient presented with a sore throat.
12. VOMIT: whether the patient presented with vomiting.

### 2.3. SARS-CoV-2 Lineages and Variants in São Paulo

Due to the different dynamics of COVID-19 lineages and variants throughout Brazil, it is important to emphasise the specific scenario of São Paulo.

**Figure 1:**
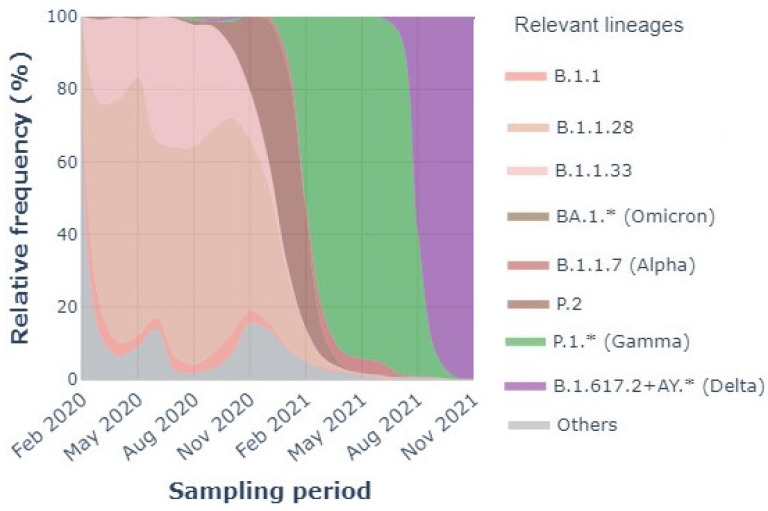
SARS-CoV-2 relevant lineages in São Paulo state (February 2020–November 2021) [21].

Figure 1 shows the lineages and variants present in 2020 and 2021. Checking was performed every three months, and the relevant lineages, i.e., the Variants of Concern (VOC), are described in the legend. As can be seen, there were four different lineages during 2020, including others, B.1.1, B.1.1.28, and B.1.1.33, with the last two being the most prevalent. The lineages overlapped during the entire year, with B.1.1.28 present in 20–80% of the population.

For 2021, the scenario is different but with significant similarity. Variant P.1 (B.1.1.28.1), also known as Gamma, was the major variant of the year; however, P.1 was derived from lineage B.1.1.28, which was the most prevalent in 2020. During NOV2020-FEB2021, the presence of variant P.2 (B.1.1.28.2), known as Zeta, derived from B.1.1.28, but as it was different from the lineage and P.1, the variant was noted. P.2 is a Variant of Interest (VOI) [22].

SARS-CoV-2 symptoms are easily confused with a cold or flu in mild cases; however, in moderate cases, the disease is strongly associated with respiratory symptoms, mainly dyspnoea, also known as shortness of breath [23]. Among the B.1.1.33 lineage, the most related symptoms are headache, rhinorrhea, cough, fatigue, myalgia, and diarrhoea [24,25], while the symptoms of B.1.1.28 are fever, followed by sore throat and cough, myalgia, fatigue, diarrhoea, and vomiting, which are also symptoms of P.1 and P.2 variants [26,27,28,29].

### 2.4. Association Rule Mining

Association rule mining (ARM) is a rule-based method that aims to extract relations (associations/links/dependencies) between variables in large datasets. ARM was first introduced by Agrawal et al. [30], where the technique was used on sales data to discover rules that predict the occurrence of an item based on the occurrence of other items [12,13]. In a clinical context, a rule that associates symptoms (or diseases) can be expressed as X→Y, where *X* and *Y* are disjoint sets of symptoms (or diseases); that is, X∩Y=⌀ [31]. *X* is called the rule’s antecedent, while *Y* is called the rule’s consequent; that is, an antecedent is an item found within the dataset and a consequent is an item that appears because of the occurrence of the antecedent [11,13].

To measure the effectiveness of the rules, one can use different parameters. In the experiments, each patient was considered as a transaction, and the rules were formed based on the support, confidence, and lift parameters. Below is a description of the parameters [12,32].

- Support measures the frequency of items in the datasets, that is, how often the rule items have been repeated in the dataset. A rule with high support implies that a significant portion of the dataset applies to the rule.
  (1) $$Support\left(X\to Y\right)=\frac{\mathrm{Number}\mathrm{of}\mathrm{transaction}\mathrm{containing}\mathrm{both}X\mathrm{and}Y}{\mathrm{Total}\mathrm{number}\mathrm{of}\mathrm{transactions}\mathrm{in}\mathrm{dataset}}$$

- Confidence measures how often the rule is true. It measures the percentage of rules containing all items in *X* and *Y* from that rules containing items in *X* alone, that is, the percentage of transactions that contain antecedents that also contain outcomes.
  (2) $$Confidence\left(X\to Y\right)=\frac{Support(X\cup Y)}{Support(X)}$$

- Lift is a important parameter used to define the correlation between *X* and *Y*.
  (3) $$Lift\left(X\to Y\right)=\frac{Support(X\cup Y)}{Support(X)*Support(Y)}$$
  – If the value of the lift is equal to 1, it means that *X* and *Y* are independent of each other, and no rule can be drawn from it.
  – If the value of lift is greater than one, it means that *X* and *Y* are dependent on each other.
  – If the value of lift is less then one, it means that *X* and *Y* are substitute of each other, defining a negative relationship, where the presence of one can have a negative impact on the other.

#### Apriori Algorithm

The Apriori algorithm is an ARM method that was first proposed by Agrawal et al. [33]. The algorithm is designed to find all frequent item sets in a dataset. The Apriori algorithm consists of the following main steps [34,35]:

- Set the minimum support threshold—to determine the minimum frequency that an itemset must have to be considered “frequent”;
- Identify frequent individual items: count the occurrences of each item to find those that meet the minimum support threshold;
- Generate candidate itemsets of size 2—form pairs from the frequent items identified;
- Exclude non-frequent itemsets: discard any itemsets that do not meet the support threshold;
- Generate larger itemsets—combine frequent itemsets to create those of sizes 3, 4, and beyond;
- Repeat the excluding process: continue eliminating item sets that fall below the support threshold;
- Iterate until no new frequent item sets are generated;
- Generate association rules: derive rules that express the relationships between items and calculate measures to evaluate their strength and significance.

The algorithm ends when no more successful extensions can be identified;

The experiments were implemented in Python [36] in the Apriori algorithm; this was carried out following the literature [11,12], using a confidence threshold of 0.9, or 90%, and a lift greater than 1. A minimum support threshold of 0.01 was used.

## 3. Results

In this section, the results obtained using the Apriori algorithm for July 2020 and March 2021 are presented, during which the highest number of SARS infection cases was recorded in São Paulo. The Brazilian public health system classifies SARS cases into categories, including *SARS caused by influenza*, *SARS caused by another respiratory virus*, *SARS caused by another etiological agent*, *SARS caused by COVID-19*, and *unspecified SARS*. The latter refers to cases in which no alternative etiological agent is identified, making it impossible to collect or process clinical samples for laboratory diagnosis or to confirm via clinical–epidemiological criteria, clinical imaging, or clinical diagnosis. However, it is not possible to separate samples that are negative for COVID-19 from other sample types.

Thus, the experiments aimed to analyse the possible similarities between the symptoms rule cases of unspecified SARS and SARS-CoV-2. For this purpose, the mentioned periods of peak infection cases for individuals with positive diagnoses and the same period for individuals with unspecified diagnoses were analysed.

The percentages of positive and unspecified individuals who reported symptoms were analysed. The pie charts present the percentage of the presence of each symptom in positive and unspecified individuals during the cited periods, noting that the percentages do not sum to one hundred percent as an individual may exhibit at least two symptoms simultaneously.

**Figure 2:**
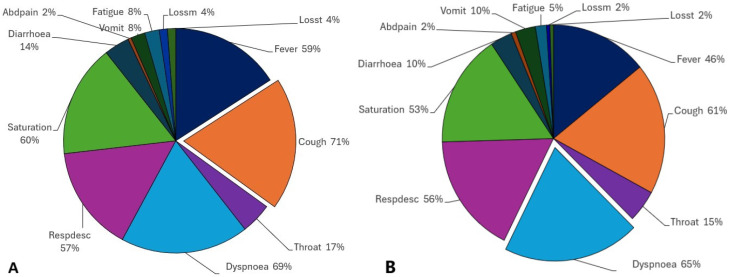
Positive tested individuals (**A**) and unspecified individuals (**B**) that presented with symptoms in July 2020.

Observing the positive population of July 2020 (Figure 2A), we see that the most common symptom was cough (71%), followed by other correlated respiratory symptoms, such as dyspnoea (69%), saturation (60%), and respiratory discomfort (57%). Then, flu-like symptoms, such as fever (59%) and sore throat (17%), appeared, in addition to diarrhoea (14%), vomiting (8%), and abdominal pain (2%). Fatigue (which may also consist of myalgia, with 8%) was followed by loss of smell and taste (4%).

However, when comparing positive and unspecified SARS (Figure 2B) diagnosed populations, a similar scenario is observed. First, dyspnoea was the most reported symptom (65%), followed by cough (61%), respiratory discomfort (56%), and saturation (53%). Fever, as before, is a flu-like symptom that was significantly present (46%), together with a sore throat (15%). Diarrhoea and vomiting were reported by 10% of the population, and abdominal pain was reported by 2%. Fatigue was present in fewer individuals than before (5%), as well as loss of smell and taste (2%).

**Figure 3:**
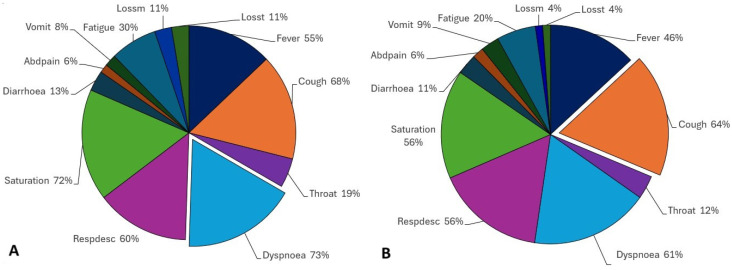
(**A**) Positive tested individuals and (**B**) unspecified individuals that presented with symptoms in March 2021.

In March 2021 (Figure 3), different from before, in positive individuals, the most common symptom was not a flu-like symptom, but a respiratory correlated symptom, dyspnoea (73%), followed by saturation (72%), with cough being the third most common symptom (68%), followed by respiratory discomfort (60%). Fever was present in 55% of individuals and a sore throat in 19% of them. Diarrhoea was the most common gastrointestinal symptom (13%), vomiting was present in 8%, and abdominal pain was present in 6% of the positively diagnosed population. Here, fatigue is a considerable symptom, reported by 30% of the individuals, and a loss of smell and taste is more present than before, with an 11% prevalence in both cases.

For the unspecified SARS population, the scenario mirrors that of positive patients, albeit with some differences. The most commonly reported symptom is cough, present in 64% of individuals, closely followed by dyspnoea, reported by almost the same percentage (61%). Respiratory discomfort and saturation were reported by 56% of the individuals. Other flu-like symptoms, such as fever and sore throat, were noted by 46% and 12% of individuals, respectively. Gastrointestinal symptoms were also reported in similar proportions. Diarrhoea affected 11% of the population, while abdominal pain was present in the same proportion as in positive cases (6%). Vomiting occurred in 9% of individuals, while fatigue was reported by fewer individuals compared to positive cases (20%). Loss of smell and taste were each reported by 4% of the population.

By analysing both positive and unspecified groups in both periods, we can point out some significant similarities. First, we noted that the most reported symptoms were respiratory symptoms, among them being cough, which was a consequence of almost all rules, and dyspnoea, a characterising symptom of moderate COVID-19; these appeared in more than half of the positive and unspecified populations. Second, the remaining symptoms, that is, flu-like ones, gastrointestinal symptoms, fatigue, and loss of taste and smell present a very similar frequency as well as a pattern in the order of symptom reporting. After respiratory ones, for both populations, the following symptoms are always fever and a sore throat, then diarrhoea, vomiting, abdominal pain, fatigue, and loss of smell and taste, with these being the last eight common symptoms in mild cases; however, in our experiments, they were present in positive and unspecified individuals.

Due to the comparable frequency of symptoms between the two groups, we delved into the persistence of these resemblances by contrasting symptom patterns among individuals diagnosed with COVID-19 and those with unspecified SARS diagnoses.

### Symptoms Rules

Using the Apriori algorithm to extract symptom rules, 67 positive and 29 unspecified rules were obtained for July 2020. For March 2021, 1574 positive and 132 unspecified rules were extracted. Table 1, Table 2, Table 3 and Table 4 present the top 10 rules for both positive and unspecified populations for both periods. Additionally, an explanation of the top rules for each group is provided.

Upon initial observation, some general characteristics of both periods and groups can be identified. Throat symptoms are present in almost all rules, along with respiratory symptoms. Every rule includes at least one throat, respiratory, or gastrointestinal symptom. All outcomes of the rules are respiratory symptoms, with the majority being cough. In July 2020, all outcomes in the positive rules were cough, which is an antecedent symptom in just one rule for all periods. In March, the presence of loss of smell and taste, symptoms that were not prominent in the July rules, was observed.

1. Rule 1 (COVID-19): throat, dyspnoea → cough

- Support: 0.10.
- Confidence: 0.90.
- Lift: 1.27.

Rule 1 (positive) suggests that when a patient has throat and dyspnoea symptoms, there is a 90% chance that they will also experience cough. The lift indicates a positive association between throat, dyspnoea, and cough, meaning that the occurrence of antecedents increases the probability of coughing.

2. Rule 1 (unspecified SARS): saturation, fatigue, dyspnoea → respdesc

- Support: 0.02.
- Confidence: 0.90.
- Lift: 1.60.

Rule 1 (unspecified) suggests that when a patient has saturation, fatigue, and dyspnoea symptoms, there is a 90% chance that they will also experience respiratory discomfort. The lift indicates a positive association between saturation, fatigue, dyspnoea, and respiratory discomfort, meaning that the occurrence of antecedents increases the probability of respiratory discomfort.

Observing both groups, significant similar patterns between the positive and unspecified rule groups can be seen. First, in July 2020, the same rule was noted in both the positive and unspecified groups: (diarrhoea, throat, dyspnoea) → cough. It is also noticeable that some unspecified rules are composed of positive rules, such as the unspecified rule (saturation, throat, dyspnoea) → cough, which is a combination of the positive rules (throat, dyspnoea) → cough and (saturation, throat) → cough. It is also worth mentioning the unspecified rule (respdesc, diarrhoea, fever, throat) → cough, which is the combination of the positive rules (diarrhoea, respdesc, throat) → cough and (fever, throat, dyspnoea) → cough. Another one is the unspecified rule (throat, respdesc, diarrhoea, dyspnoea) → cough, which is the combination of the positive rules (respdesc, throat, dyspnoea) → cough and (throat, dyspnoea) → cough. It can also be seen that the unspecified rules are a permutation of gastrointestinal symptoms on positive rules, such as the unspecified rule (saturation, vomit, throat) → cough, which is the same as the positive rule (saturation, diarrhoea, throat) → cough, but just changing the vomiting for diarrhoea symptoms.

1. Rule 1 (COVID-19): fatigue, respdesc → dyspnoea

- Support: 0.20.
- Confidence: 0.90.
- Lift: 1.23.

Rule 1 (positive) suggests that when a patient has symptoms of fatigue and respiratory discomfort, there is a 90% chance that they will also experience dyspnoea. The lift indicates a positive association between fatigue, respiratory discomfort, and dyspnoea, indicating that the occurrence of antecedents increases the probability of dyspnoea.

2. Rule 1 (unspecified SARS): fever, throat, dyspnoea → cough

- Support: 0.04.
- Confidence: 0.91.
- Lift: 1.43.

Rule 1 (unspecified) suggests that when a patient has fever, throat, and dyspnoea symptoms, there is a 91% chance that they will also experience a cough. The lift indicates a positive association between fever, throat, dyspnoea, and cough, meaning that the occurrence of antecedents increases the probability of cough.

For March, a similar dynamic is observed in unspecified and positive rules as in the previous period. It can be mentioned that the unspecified rule (fatigue, throat, dyspnoea) → cough, which is the combination of positive rules (fatigue, throat) → cough and (fatigue, respdesc) → dyspnoea, substitutes the respiratory symptom of dyspnoea for respiratory discomfort. It can also be seen that the unspecified rule (abdpain, respdesc, diarrhoea) → cough, which is the same as the positive rule (abdpain, throat, diarrhoea) → cough, only changes the throat symptoms for respiratory discomfort symptoms. Similarly, the unspecified rule (diarrhoea, throat, dyspnoea) → cough is the same as the positive rule mentioned before, changing dyspnoea to abdominal pain symptoms.

It has been previously noted that the symptom rules for positive and unspecified rules are very similar. In 2020, the COVID-19 lineages overlapped throughout the entire year, and in 2021, the primary variant was derived from the 2020 lineage. Due to these significant and apparent symptom patterns between positive and unspecified diagnosed rules, we decided to compare them by checking whether they were the same. For this, checks were performed throughout the entire year for both periods (2020 and 2021) using the top five rules of each group, according to its support.

**Figure 4:**
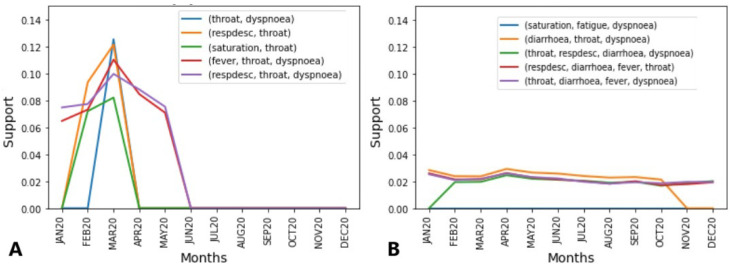
(**A**) Top 5 symptom rules of positive individuals found in unspecified individuals in July 2020. (**B**) Top 5 symptom rules of unspecified individuals found in positive individuals in July 2020.

For both periods, in Figure 4A, the box shows the top five COVID-19 rules found in the unspecified SARS rules set, that is, the data plotted. In Figure 4B, the opposite is shown, and the top five unspecified SARS rules are exhibited in the text box but the plotted data refer to the rules found on the COVID-19 rules set.

Initially, as shown in Figure 4A, it was observed that the most frequent rule (14%) also includes dyspnoea, that is, as mentioned before, a symptom strong linked with moderate COVID-19 cases. The second top rule also includes throat symptoms, in addition to respiratory distress, which reached similar support (12% and 14%), and is also a symptom linked with moderate cases of COVID-19. The third rule is composed of throat and saturation symptoms, indicators of respiratory distress and even dyspnoea. The fourth and fifth rules are similar to the previous ones, but both were not present in JUN20, and the first three are not present earlier, APR20. They also have in common the fact that their highest peak was reached in MAR20.

The abrupt disappearance of cases can be explained by the fact that the first case of COVID-19 was officially recognised in March 2020; thus, the SARS-CoV-2 cases from JAN20-FEB20, initially considered unspecified SARS or even other diseases, began to be classified as COVID-19, which can explain the high rule support until March 2020.

In Figure 4B, we can see a similar behaviour concerning the symptoms that composed the rules, with the significant presence of dyspnoea, saturation, and respiratory distress, but also mild COVID-19 symptoms, such as a sore throat and fever, as well as the presence of diarrhoea in three of the five rules. Diarrhoea as well as a sore throat and fever are linked with mild COVID-19 cases. All the rules showed the same behaviour for almost the entire year, hanging over 2% and 4%. As mentioned before, the same lineages overlapped each month, which can explain their presence in almost all months. This behaviour indicates that regardless of the lineage, the COVID-19 rules were consistently present among the unspecified SARS symptom rules.

**Figure 5:**
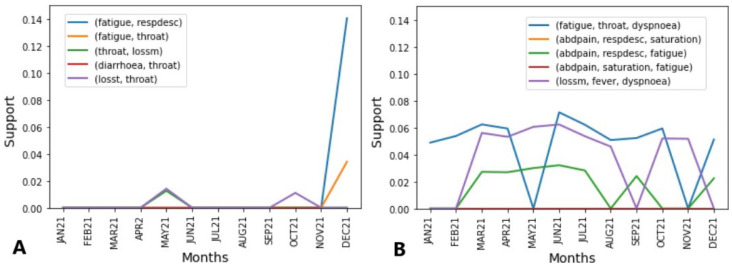
(**A**) Top 5 symptoms rules of positive individuals found in unspecified individuals on March 2021. (**B**) Top 5 symptoms rules of unspecified individuals found in positive tested individuals on March 2021.

Observing Figure 5A, a different scenario for 2020 can be seen. The data refer to the unspecified SARS symptoms rules set during the second year of the pandemic, and it can be noted that the COVID-19 rules were not confused with unspecified SARS, figuring in only MAY21, OCT21, NOV21, where the rule (fatigue, respdesc) → cough reached 14% support. However, different from before, two symptoms characteristic of COVID-19 appear, loss of smell and taste, as seen in three of the five top rules.

Figure 5B shows a different scenario. First, only three of the top five rules are present in the entire year, hanging over 2% and 8%. Notably, they are composed of at least fatigue which, as mentioned before, was a symptom frequently reported (30%) by COVID-19 infected individuals. It is also noted that throat symptoms are only present in the most frequent rule, as well as loss of smell, which is present in the less frequent rule. Abdominal pain symptoms are in three of them, being linked to mild cases. Here, it is noted that in comparison with 2020, the mild symptoms of a sore throat and fever, as well as diarrhoea, replace the other mild symptoms, such as abdominal pain, that were not frequent symptoms communicated by COVID-19 and unspecified SARS individuals; however, it can be explained by the fact that even with overlapping lineages, gastrointestinal symptoms are more related with the P.1. variant, which was predominant during May–November 2021.

## 4. Discussion

This analysis was based on two main conclusions. First, in 2020 and 2021, according to the databases provided by the Brazilian Ministry of Health, there was no significant circulation of another virus that caused SARS than SARS-CoV-2. Secondly, during the pandemic, there was a notorious shortage of resources, especially RT-PCR tests, which are the gold standard for diagnosing COVID-19. This scarcity, consistent with findings from other studies, may have led to the under reporting of cases [37,38,39,40]. Consequently, unspecified SARS labelling raised suspicion that some SARS-CoV-2 infections might not have been identified as COVID-19.

Our first finding involved the occurrence of moderate COVID-19 symptoms in very similar percentages in individuals labelled as having unspecified SARS, notably dyspnoea (shortness of breath), a symptom strongly linked with moderate COVID-19 cases and symptoms linked with mild cases, such as gastrointestinal symptoms. Another finding was that the symptom rules from both sets predominantly have the same symptom, cough. In addition, unspecified SARS rules seem to be a composition of COVID-19 rules, significantly differing from SARS-CoV-2 rules in the exchange of one symptom for another from the same category, such as diarrhoea for abdominal pain, with both being gastrointestinal symptoms. In addition, there is a consistent presence of the most frequent COVID-19 symptom rules among patients with unspecified SARS in 2020 and 2021.

Thus, in terms of health policies, the analysis of association rules can facilitate the identification of symptom behaviour patterns and characteristic symptoms of diseases, especially in pandemic scenarios. It can also highlight signs of under reporting, as inadequate understanding of disease spread can affect different aspects of public health. Additionally, isolation recommendations could be made even without laboratory tests by alerting patients to the similarity of their symptoms to those diagnosed with COVID-19.

The limitations of this study primarily stem from the spatial constraints of the data. Given Brazil’s vast geographic size, COVID-19 exhibited different behaviours across various regions of the country. Consequently, we believe it is not feasible to generalise conclusions from an analysis encompassing the entire country. Therefore, we selected São Paulo, a state that proportionally represents the country’s population. São Paulo is the most populous state in Brazil [41], with it being the largest metropolis in South America and having significant national and international tourist flows. Initially, selecting just one state may seem unrepresentative, but the dynamic movement of people into, out of, and within São Paulo, especially in its capital, provides a unique scenario for examining possible interactions between different COVID-19 strains and variants. Another limitation pertains to the number of association rules generated for both groups based on the chosen parameters, particularly in 2021. For effective visualisation and comprehension, we selected a reduced number of rules. Nonetheless, the selected rules were the most frequent in both sets, enabling us to analyse the most apparent behaviours.

## 5. Conclusions

In this study, the use of the Apriori algorithm, an association rule mining technique, was proposed to investigate and analyse similarities between COVID-19 and unspecified SARS symptom rules.

Our first finding involved the occurrence of moderate COVID-19 symptoms in individuals with unspecified SARS, notably dyspnoea (shortness of breath), which appeared in cases of COVID-19 at similar rates as in individuals with unspecified SARS. In addition, gastrointestinal symptoms, such as vomiting and diarrhoea, together with flu-like symptoms, which are frequently linked to mild COVID-19 cases, are also present in unspecified SARS individuals; loss of smell and taste, specific symptoms of COVID-19, was present in unspecified SARS, being diagnosed at similar rates to COVID-19. In addition, we noted that most of the present symptom categories, respiratory symptoms, and gastrointestinal symptoms, were prevalent symptoms of the variants in circulation during the study period.

Analysing the association rules, a strong indication of similarity was found in that both COVID-19 and the unspecified SARS rule’s consequent are predominantly the same symptom, cough. Furthermore, unspecified SARS rules shorten the combination of respiratory, gastrointestinal, and flu-like symptoms, with the last two groups of symptoms being linked to mild cases. Unspecified rules can be decomposed into COVID-19 rules, differing by exchanging one symptom from another in the category, such as changing vomiting for diarrhoea, for example, making the set of unspecified rules a subset of COVID-19 rules. Another important finding was the consistent presence of COVID-19 rules among the unspecified SARS cases during the entire analysed period, as lineages and COVID-19 variants overlapped during 2020 and 2021. Moreover, in the first two years of the pandemic, Brazil experienced a lack of correct health supplies, especially RT-PCR tests and COVID-19 diagnosis laboratory examinations. In addition, from the analysed data, it is possible to deduce that there was no significant circulation of other respiratory viruses or etiological agents that caused SARS.

By analysing the findings in the face of the health situation in these periods, we conjecture a scenario where mild cases were directed to the health system but not properly diagnosed, and due to similar symptoms to those of SARS-CoV-2, such cases were labelled as unspecified SARS.

Consequently, this scenario leads us to surmise that a considerable proportion of individuals categorised as having unspecified SARS could be infected with SARS-CoV-2. Thus, to improve this type of investigation, it is important to consider techniques that seek to improve performance while considering the issue of possible mislabelling. As an improvement to this study, a machine learning technique such as weak labelling [42] should be considered.

## Figures and Tables

**Table 1:** Top 10 rules for COVID-19 diagnosed individuals in July 2020 period.

	July 2020 COVID-19				
	**Antecedent**	**Consequent**	**Support**	**Confidence**	**Lift**
**Rule 1**	(throat, dyspnoea)	(cough)	0.10	0.90	1.27
**Rule 2**	(respdesc, throat)	(cough)	0.09	0.90	1.27
**Rule 3**	(saturation, throat)	(cough)	0.09	0.90	1.27
**Rule 4**	(fever, throat, dyspnoea)	(cough)	0.08	0.91	1.29
**Rule 5**	(respdesc, throat, dyspnoea)	(cough)	0.08	0.91	1.29
**Rule 6**	(diarrhoea, throat, fever)	(cough)	0.02	0.91	1.28
**Rule 7**	(diarrhoea, respdesc, throat)	(cough)	0.02	0.92	1.29
**Rule 8**	(diarrhoea, throat, dyspnoea)	(cough)	0.02	0.91	1.29
**Rule 9**	(saturation, diarrhoea, throat)	(cough)	0.02	0.91	1.28
**Rule 10**	(fatigue, throat)	(cough)	0.01	0.90	1.27

**Table 2:** Top 10 rules for unspecified SARS diagnosed individuals in July 2020 period.

	July 2020 Unspecified SARS				
	**Antecedent**	**Consequent**	**Support**	**Confidence**	**Lift**
**Rule 1**	(saturation, fatigue, dyspnoea)	(respdesc)	0.02	0.90	1.60
**Rule 2**	(diarrhoea, throat, dyspnoea)	(cough)	0.01	0.91	1.40
**Rule 3**	(throat, respdesc, diarrhoea, dyspnoea)	(cough)	0.01	0.91	1.51
**Rule 4**	(respdesc, diarrhoea, fever, throat)	(cough)	0.01	0.91	1.48
**Rule 5**	(throat, diarrhoea, fever, dyspnoea)	(cough)	0.01	0.91	1.50
**Rule 6**	(throat, saturation, diarrhoea, dyspnoea)	(cough)	0.01	0.91	1.50
**Rule 7**	(saturation, vomit, throat)	(cough)	0.01	0.90	1.47
**Rule 8**	(saturation, throat, dyspnoea)	(cough)	0.06	0.90	1.46
**Rule 9**	(respdesc, saturation, throat)	(cough)	0.05	0.90	1.46
**Rule 10**	(cough, respdesc, diarrhoea, saturation)	(dyspnoea)	0.02	0.90	1.39

**Table 3:** Top 10 rules for COVID-19 diagnosed individuals in March 2021 period.

	March 2021 COVID-19				
	**Antecedent**	**Consequent**	**Support**	**Confidence**	**Lift**
**Rule 1**	(fatigue, respdesc)	(dyspnoea)	0.20	0.90	1.23
**Rule 2**	(fatigue. throat)	(cough)	0.07	0.90	1.32
**Rule 3**	(throat, lossm)	(cough)	0.03	0.91	1.34
**Rule 4**	(diarrhoea, throat)	(cough)	0.03	0.91	1.34
**Rule 5**	(losst, throat)	(cough)	0.03	0.91	1.33
**Rule 6**	(abdpain, throat)	(cough)	0.02	0.92	1.35
**Rule 7**	(vomit, throat)	(cough)	0.02	0.90	1.33
**Rule 8**	(abdpain, lossm)	(cough)	0.01	0.90	1.32
**Rule 9**	(abdpain, diarrhoea, fever)	(cough)	0.01	0.90	1.32
**Rule 10**	(abdpain, diarrhoea, throat)	(cough)	0.01	0.94	1.38

**Table 4:** Top 10 rules for unspecified SARS diagnosed individuals in March 2021 period.

	March 2021 Unspecified SARS				
	**Antecedent**	**Consequent**	**Support**	**Confidence**	**Lift**
**Rule 1**	(fever, throat, dyspnoea)	(cough)	0.04	0.91	1.43
**Rule 2**	(fatigue, throat, dyspnoea)	(cough)	0.02	0.91	1.43
**Rule 3**	(abdpain, respdesc, saturation)	(dyspnoea)	0.02	0.92	1.50
**Rule 4**	(abdpain, respdesc, fatigue)	(dyspnoea)	0.01	0.90	1.48
**Rule 5**	(abdpain, saturation, fatigue)	(dyspnoea)	0.01	0.91	1.48
**Rule 6**	(lossm, fever, dyspnoea)	(cough)	0.01	0.90	1.41
**Rule 7**	(diarrhoea, throat, dyspnoea)	(cough)	0.01	0.90	1.42
**Rule 8**	(respdesc, diarrhoea, throat)	(cough)	0.01	0.90	1.42
**Rule 9**	(saturation, diarrhoea, throat)	(cough)	0.01	0.91	1.43
**Rule 10**	(abdpain, respdesc, diarrhoea)	(cough)	0.01	0.90	1.41

## Data Availability

The data used in this work are available in OpenDataSUS (https://opendatasus.saude.gov.br/, a database maintained by Brazil Ministry of Health (accessed on 15 January 2024). The code developed to perform the experiments is available at https://github.com/JuhCrln/association_rules_covid.git (accessed on 21 March 2024).

## Abbreviations

The following abbreviations are used in this manuscript:

WHO	World Health Organization;
SARS	Severe acute respiratory syndrome;
SARS-CoV-2	Severe acute respiratory syndrome coronavirus 2;
ARM	Association rule mining;
SP	São Paulo;
VOC	Variant of Concern;
VOI	Variant of Interest.
